# Immunomodulation by glucocorticoid-induced leucine zipper in macrophages: enhanced phagocytosis, protection from pyroptosis, and altered mitochondrial function

**DOI:** 10.3389/fimmu.2024.1396827

**Published:** 2024-05-23

**Authors:** Thierry M. Legroux, Hanna S. Schymik, Gilles Gasparoni, Saeed Mohammadi, Jörn Walter, Claude Libert, Britta Diesel, Jessica Hoppstädter, Alexandra K. Kiemer

**Affiliations:** ^1^ Department of Pharmacy, Pharmaceutical Biology, Saarland University, Saarbrücken, Germany; ^2^ Department of Genetics, Saarland University, Saarbrücken, Germany; ^3^ Natural and Medical Sciences Research Center, University of Nizwa, Nizwa, Oman; ^4^ Flanders Institute for Biotechnology (VIB) Center for Inflammation Research, Ghent, Belgium; ^5^ Department of Biomedical Molecular Biology, Ghent University, Ghent, Belgium

**Keywords:** RNA-seq, immunometabolism, extracellular matrix, lipopolysaccharide (LPS), reactive oxygen species (ROS), iNOS

## Abstract

Glucocorticoids, which have long served as fundamental therapeutics for diverse inflammatory conditions, are still widely used, despite associated side effects limiting their long-term use. Among their key mediators is glucocorticoid-induced leucine zipper (GILZ), recognized for its anti-inflammatory and immunosuppressive properties. Here, we explore the immunomodulatory effects of GILZ in macrophages through transcriptomic analysis and functional assays. Bulk RNA sequencing of GILZ knockout and GILZ-overexpressing macrophages revealed significant alterations in gene expression profiles, particularly impacting pathways associated with the inflammatory response, phagocytosis, cell death, mitochondrial function, and extracellular structure organization activity. GILZ-overexpression enhances phagocytic and antibacterial activity against *Salmonella typhimurium* and *Escherichia coli*, potentially mediated by increased nitric oxide production. In addition, GILZ protects macrophages from pyroptotic cell death, as indicated by a reduced production of reactive oxygen species (ROS) in GILZ transgenic macrophages. In contrast, GILZ KO macrophages produced more ROS, suggesting a regulatory role of GILZ in ROS-dependent pathways. Additionally, GILZ overexpression leads to decreased mitochondrial respiration and heightened matrix metalloproteinase activity, suggesting its involvement in tissue remodeling processes. These findings underscore the multifaceted role of GILZ in modulating macrophage functions and its potential as a therapeutic target for inflammatory disorders, offering insights into the development of novel therapeutic strategies aimed at optimizing the benefits of glucocorticoid therapy while minimizing adverse effects.

## 1 Introduction

For over 60 years, glucocorticoids (GCs) have been a cornerstone in therapeutic strategies for their potent anti-inflammatory and immunosuppressive effects, addressing a spectrum of conditions such as rheumatic diseases, inflammatory bowel disease (IBD), autoimmune disorders, chronic inflammatory diseases, asthma, and cancer (1). Despite the emergence of more targeted biological therapies, GCs persist as widely utilized agents. In autoimmune and chronic inflammatory conditions, they are favored for providing symptomatic relief (2), and in the case of IBD, they serve as a fundamental treatment for moderate-to-severe cases (3). Additionally, in the oncological context, GCs play a pivotal role in managing cancer-related symptoms and addressing side effects of anti-cancer therapies and immune-related adverse events (4).

The use of GC therapy, despite its efficacy in mitigating inflammation and immune activation, is often constrained by a spectrum of side effects, frequently restricting its long-term application. These include musculoskeletal, gastrointestinal, cardiovascular, endocrine, neuropsychiatric, dermatologic, ocular, and immunologic issues (5). Of particular concern are the metabolic side effects, such as central adiposity, dyslipidemia, insulin resistance, and glucose intolerance, which can lead to diabetes (6). Other common side effects include weight gain, skin thinning, sleep disturbance, and neuropsychiatric disorders (7).

To optimize the benefits of GC therapy while minimizing adverse effects, several strategies are being explored. Metformin is being investigated as a promising drug for preventing metabolic side effects during systemic GC treatment (8). Targeted delivery of GCs to diseased tissues using antibody-glucocorticoid conjugates is being explored as a therapeutic alternative to overcome systemic side effects (9). In addition, the development of selective dimerizing GC-receptor agonists or modulators (SEDIGRAMs) aims to capitalize on specificities within the GC signaling pathway, potentially mitigating systemic side effects associated with monomeric activity (10).

GCs exert their actions on cells through a variety of mechanisms, including genomic and non-genomic pathways, tissue-specific effects, and interactions with other signaling pathways (11). Upon binding to the glucocorticoid receptor (GR) in the cytoplasm, the classical mechanism involves the hormone receptor complex modulating gene expression either positively or negatively by directly binding to GREs located in the promoter regions of target genes (12). The GR exhibits ubiquitous expression across cell types, thereby influencing the functionality of practically all immune cells (13). Approximately 20–30% of genes are deemed responsive to the GR, with the glucocorticoid-induced leucine zipper (GILZ, gene name TSC22D3) being among the earliest targets of GR, making it a promising GC-induced downstream effector molecule (10, 14, 15).

GILZ has been identified as a key mediator of the anti-inflammatory and immunosuppressive effects of GCs (16). GILZ is strongly upregulated by GCs and plays a crucial role in controlling major activities of T cells (17), B cells (18), MSCs (19), neutrophils (20), and macrophages (21).

Shortly following the characterization of GILZ in immune cells, the consideration of its therapeutic application emerged (22). Selective upregulation of GILZ and the use of recombinant GILZ protein have emerged as promising therapeutic approaches in diseases like inflammatory bowel disease (IBD), rheumatoid arthritis, psoriasis, sepsis, and diabetic retinopathy (2, 23–28). Leveraging its anti-inflammatory properties, GILZ holds the potential for therapeutic interventions in these conditions.

In macrophages, GILZ has been implicated in mediating typical GC effects, such as the regulation of macrophage activation (29). GCs aside, GILZ has demonstrated beneficial properties in combating inflammatory diseases. Transgenic mice that overexpress GILZ have been found to be more resistant to spinal cord injury, while GILZ knockout mice develop worse inflammatory conditions (30, 31). Furthermore, studies have demonstrated that selective GILZ overexpression in macrophages improves outcomes in septic animals by limiting systemic inflammation while increasing bacterial clearance (32, 33). The administration of a cell-permeable GILZ fusion protein (TAT-GILZ) facilitated the accelerated resolution of bacteria-induced pneumonia and peritonitis in mice, further underscoring the therapeutic potential of GILZ in modulating macrophage-mediated inflammation (34, 35).

Understanding the role of GILZ in macrophages and its potential therapeutic applications is crucial for advancing precision medicine in inflammatory disorders. In this context, our study employs RNA sequencing to investigate gene expression in GILZ knockout (KO) and GILZ-overexpressing (TG) macrophages (murine bone marrow-derived macrophages, BMMs), aiming to uncover the molecular mechanisms underlying GILZ-mediated effects and its therapeutic potential.

Here, we provide evidence for the immunomodulatory effects of GILZ overexpression in macrophages, demonstrating enhanced antibacterial activity, protection from pyroptosis, and altered mitochondrial function. Additionally, our findings reveal a significant impact on Matrix metalloproteinase (MMP) activity, suggesting a role for GILZ in influencing extracellular matrix remodeling and wound healing processes.

## 2 Materials and methods

### 2.1 Materials

RPMI-1640 cell culture media (#R0833), 200 mM L-glutamine (#G7513), 1% penicillin/streptomycin (#P4333), Accutase (#A6964), mitomycin C (#10107409001), antimycin A from *Streptomyces* sp. (#A8674), gentamicin (#G1397), dihydroethidium (DHE) (#37291), and gelatin type A (#G1890) were from Sigma-Aldrich. FBS (#P040–37500) and Panexin BMM Serum Substitute (#P04–951SA2) were obtained from PAN Biotech. Ultrapure lipopolysaccharide (LPS) from *E. coli* K12 (#tlrl-peklps), and ATP (#tlrl-atpl) were purchased from Invivogen. TE buffer (#A0386.1000), and DMSO (#A3672-0250) were from AppliChem, M-CSF (#130–101-705) from Miltenyi Biotech, and mouse recombinant IFN-γ (#87389.100) from Biomol. Primers for qRT-PCR were purchased from Eurofins Genomics. Other chemicals were obtained from either Sigma-Aldrich or Carl Roth unless stated otherwise.

### 2.2 Mice

Animal housing and all experimental procedures were approved by the local animal welfare committee (AZ 2.4.1.1). C57BL/6J mice were housed in individually ventilated cages in a temperature and humidity-controlled room (22–24°C, 45–65 relative humidity) and a 12 h light/dark cycle. Water and food were provided *ad libitum*. Mice were age and sex-matched within experimental sets. B6.129P2-Lyz2^tm1(cre)Ifo^/J mice with Cre recombinase expression under the endogenous *Lyz2* promotor of the myeloid cell lineage were considered *wild-type* (WT). Crossing these animals with mice bearing loxP sites up and downstream of *Gilz* exon 6 resulted in a myeloid-specific *knockout* of GILZ (B6.129P2-Tsc22d3^f/f^ Lyz2^tm1(cre)Ifo^/J, GILZ KO). Both strains were described previously (36). In addition, mice bearing a *Tsc22d3/Gilz-1* cDNA knock-in under the control of the ROSA26 promoter preceded by a loxP-flanked stop cassette (37) were crossed with B6.129P2-Lyz2^tm1(cre)Ifo^/J mice, resulting in myeloid-specific GILZ overexpression. These animals (BL6Tsc22d3^Rosa26EGFPtg^Lyz2^tm1(cre)lfo^/J) are designated as GILZ-overexpressing or transgenic (GILZ TG).

### 2.3 Cell culture conditions

Cells were cultured in a humidified incubator at 37°C and 5% CO_2_ in RPMI-1640 cell culture media supplemented with 10% FBS, 1% 200 mM L-glutamine, and 100 units/ml penicillin/streptomycin (P/S) unless stated otherwise.

### 2.4 Generation and treatment of BMMs

BMMs were obtained from 10- to 23-week-old mice as described previously (38). Mice were sacrificed, and femurs and tibias were removed. The bone marrow was flushed out with cell culture medium using a 27G cannula and passed through a 100 µm cell strainer before erythrocyte lysis in hypotonic buffer (155 mM NH_4_Cl, 10 mM KHCO_3_, 1 mM Na_2_EDTA) at 37°C for 3 min. Cells were either resuspended in FBS with 10% DMSO for cryopreservation or kept in cell culture medium supplemented with M-CSF (50 ng/ml) for differentiation to BMMs (BMM medium). Cryopreserved cells were cooled to -80°C using the MrFrosty™ device (Thermo Scientific, #5100-0001) and transferred into liquid nitrogen for long-term storage. Bone marrow cells were incubated overnight in 30 ml of BMM medium in a T75 flask. On the next day, non-adherent cells were transferred to a fresh cell culture flask and incubated for 5 more days in 45 ml of BMM medium in a T175 flask. Subsequently, cells were washed once with PBS, detached with Accutase, and counted using the LUNA-FL™ Automated Fluorescence Cell Counter (Logos Biosystems) with Acridine Orange/Propidium Iodide staining according to the manufacturer’s protocol. The viable cell count was used to calculate seeding densities. All cell preparations had a viability of at least 95%. The identity of BMMs was verified by flow cytometric analysis using F4/80 as a macrophage marker, with purity levels > 95% (38, 39). Approximately 20–30 million differentiated BMMs were obtained per mouse. BMMs were seeded and treated in BMM medium according to the specified conditions for each assay.

BMM-conditioned media for migration and proliferation assays were generated by incubating 10^6^ BMMs in a 6-well format with 3 ml per well of BMM medium for 24 h starting at the time of seeding. To assess MMP activity, 75,000 BMMs were incubated in a 96-well format with 150 µl of BMM media containing Panexin BMM serum substitute (PAN Biotech, #P04–951SA2) instead of FBS for 24 h, starting at the time of seeding.

### 2.5 RNA sequencing

For transcriptome analysis of BMMs from WT, GILZ KO, and GILZ TG mice, next-generation sequencing (NGS) was performed as described previously (40). 500,000 BMMs (1 ml/well) were seeded in a 12-well plate and treated for 4 h with LPS (100 ng/ml) on the next day. RNA was isolated using the High Pure RNA Isolation Kit (Roche, #11828665001) according to the manufacturer’s protocol and stored at -80°C. All RNA samples used for further analysis had an RNA integrity number > 9 according to the analysis in a 2100 Bioanalyzer (Agilent) using the RNA 6000 Nano Kit (Agilent, #5067–1513). Libraries were prepared from 500 ng RNA. Poly(A) enrichment was performed on the input total RNA using the NEBNext Poly(A) mRNA Magnetic Isolation Module (New England Biolabs, #E7490) according to the manufacturer’s instructions. The cDNA library preparation was conducted with the NEBNext Ultra Directional RNA Library Prep Kit for Illumina (New England Biolabs, #E7420) as recommended by the supplier. In brief, first- and second-strand cDNA synthesis was performed, followed by adapter ligation and PCR amplification of the final library (12 cycles). PCR cleanup was performed using Agencourt AM-Pure XP beads (Beckmann Coulter, #A63881). Libraries were sequenced for 1x 75 nt on a NextSeq500 (Illumina) sequencer.

### 2.6 RNA-seq data processing and analysis

Raw reads were demultiplexed and subjected to quality control through FastQC v0.11.2. Read processing was performed with grape-nf pipeline (v1.1.3) using Nextflow (v20.10.0) and mapped to GRC38mm10 assembly. The counts obtained after alignment were used to analyze differential expression using DESeq2 v1.40.2. Principle component analysis was performed using the CPM values of all annotated protein-coding genes. All analyses were conducted in the R programming language. DESeq2 analysis revealed differentially expressed genes (DEGs, *p* < 0.05) in the comparisons of GILZ KO vs. WT, GILZ TG vs. WT, and GILZ KO vs. GILZ TG under both untreated and LPS-treated conditions. Subsequently, TPM values of the DEGs from all three contrasts were subjected to k-Means unsupervised clustering using iDEP 1.12, independently for untreated and LPS-treated cells (41). Processed and raw data were deposited in the Gene Expression Omnibus (GEO) database under the accession code GSE254137.

### 2.7 pHrodo™ phagocytosis assay

50,000 BMMs (150 µl/well) were seeded into a 96-well plate and incubated overnight. pHrodo™ Red *S. aureus* Bioparticles™ (Thermo Scientific, #A10010) were suspended in PBS and treated in an ultrasonic water bath for 15 min at 37°C according to the manufacturer’s protocol before adding 5 µl of a 1 µg/µl dilution to each well. Real-time imaging was started immediately in the Incucyte^®^ S3 Live-Cell Analysis System (Essen BioScience) in the brightfield and red fluorescence channel (10x objective lens, 400 ms acquisition time). The phagocytic capacity was calculated as the total red object integrated intensity (RCU x µm²/Image) normalized to cell confluency [%] at the start of the assay as described previously (42).

### 2.8 *Salmonella enterica* and *Escherichia coli infection* assay

*Salmonella enterica subsp. enterica serotype Typhimurium* (NCTC^®^ 12023) (43) and *Escherichia coli* TOP10 (Invitrogen™) were cultured overnight in LB at 37°C in an orbital shaker. Bacteria were washed once in PBS and resuspended in BMM media without P/S supplementation to infect BMMs at a multiplicity of infection (MOI) of 20 with *S. typhimurium* and an MOI of 100 with *E. coli*. Bacterial cell count was determined by OD_600_ measurement assuming that an OD_600_ of 1 equates to 10^9^ colony-forming units (CFU). BMMs were seeded the day before in a 24-well plate (250,000 cells/well, 0.5 ml). Infection was synchronized by centrifuging for 5 min at 300 x *g* and maintained for 30 min. After the infection, BMMs were washed twice with PBS and cultured in cell culture media containing 100 µg/ml gentamicin for 90 min to kill extracellular bacteria, after which the gentamicin concentration was reduced to 10 µg/ml. BMMs were lysed in 1% Triton-X 100 after a total incubation time of 3 h for *E. coli* and 6 h for *S. typhimurium*. Lysis was stopped by adding LB media. The bacterial load of the infected BMMs was determined by serially diluting the lysates in PBS and plating them out on LB agar as described previously (44). Plates were incubated for 24 h at 37°C and CFUs were counted using ImageJ (Version 1.53k) (45).

### 2.9 Griess assay

Nitric oxide (NO) production was assessed by Griess assay as previously described (42). BMMs were seeded the day before in a 24-well plate (250,000 cells/well, 0.5 ml). On the next day, cells were treated with LPS (100 ng/ml) and IFN-γ (25 ng/ml) for 24 h before 100 µl of supernatant was incubated for 10 min at room temperature with 90 µl 1% sulfanilamide in 5% phosphoric acid, then 90 µl 1% N-1-naphthylethylenediamine dihydrochloride (NED) was added and incubated for 5 min until absorbance was measured at λ = 560 nm in a GloMax^®^ Discover Microplate Reader (Promega). Absolute concentrations were determined by measuring a NaNO_2_ standard curve in parallel for each assay. Data were normalized to protein contents measured by the Pierce™ BCA Protein Assay Kit according to the manufacturer’s protocol (Thermo Scientific, #23227).

### 2.10 RNA isolation, reverse transcription, and qRT-PCR

Cells were infected with *S. typhimurium* as described above and subsequently stored at -80°C until RNA was isolated using the High Pure RNA Isolation Kit (Roche, #11828665001) or the Direct-Zol RNA Miniprep Kit (ZymoResearch, #R2052) according to the manufacturer’s protocol. Reverse Transcription was performed using the High-Capacity cDNA Kit (Thermo Scientific, #4368813) according to the manufacturer’s protocol using 300–500 ng of RNA per reaction. RT products were diluted in TE buffer. qRT-PCR reactions were performed in technical duplicates or triplicates using HOT FIREPol EvaGreen qPCR Mix Plus (no ROX) (Solis Biodyne, #082500020) according to the manufacturer’s protocol as described previously (40, 46). The qRT-PCR reaction comprised 15 min at 95°C and 40 cycles of 15 s at 95°C, 20 s at 60°C, and 20 s at 72°C. Quantification was achieved by the 2^-ΔΔCq^ method. The primer sequences are depicted in **Table 1**.

**Table 1:** Primer sequences for qRT-PCR reactions.

Gene	Transcript RefSeq (NCBI)	Forward sequence (5′→3′)	Reverse sequence (5′→3′)
*Arg1*	NM_007482.3	ACAAGACAGGGCTCCTTTCAG	GGCTTATGGTTACCCTCCCG
*Arg2*	NM_009705.3	ATCCCCTCCCTGCCAATCAT	CTAGCTTCTTCTGTCCCCGA
*Nos2*	NM_010927.3	CTTCCTGGACATTACGACCC	TACTCTGAGGGCTGACACAA
*Ppia*	NM_008907.1	GCGTCTCCTTCGAGCTGTTT	CACCCTGGCACATGAATCCT

### 2.11 Real-time caspase-3/7 activity and cytotoxicity detection

50,000 BMMs (150 µl/well) were seeded in 96-well plates and incubated overnight. To induce pyroptosis, cells were treated for 4 h with LPS (100 ng/ml) and subsequently with ATP (2 mM). Real-time imaging was started immediately after LPS treatment using the Incucyte^®^ S3 Live-Cell Analysis System (Essen BioScience) in brightfield, red, and green fluorescence channels (10x objective lens, 300 ms acquisition time for the green channel and 400 ms for the red channel). Culture media was supplemented with 4 µM CellEvent™ Caspase-3/7 Green Detection Reagent (Invitrogen, #C10723) and 0.25 µM Incucyte^®^ Cytotox Red Reagent (Essen BioScience, #4632) as described previously (47). Total red and green object integrated intensities (RCU x µm²/Image) was normalized to the initial cell confluency [%] in each well. Background fluorescence at the beginning of the assay was uniformly set to 0 for all wells. Spectral unmixing was set to 1.5% removal of red signal from green as recommended by the manufacturer.

### 2.12 ELISA

500,000 BMMs (1 ml/well) were seeded in 12-well plates and incubated overnight. Cells were treated for 4 h with LPS (100 ng/ml) and subsequently for 30 min with ATP (2 mM) before supernatants were stored at -80°C. IL-1β secretion was measured by ELISA (BioLegend #432604) according to the manufacturer’s protocol. Supernatants were diluted 1:25 in ELISA assay diluent.

### 2.13 Detection of reactive oxygen species

50,000 BMMs (150 µl/well) were seeded in 96-well plates and incubated overnight. Cells were treated for 4 h with LPS (100 ng/ml). Then, culture medium was exchanged to medium containing ATP (2 mM) and 10 µM dihydroethidium (DHE). Real-time imaging was started immediately in the Incucyte^®^ S3 Live-Cell Analysis System (Essen BioScience) in the brightfield and red fluorescence channels (10x objective lens, 400 ms acquisition time) upon ATP treatment. Total ROS production was determined as the total red object integrated intensity (RCU x µm²/Image) after 30 min normalized to cell confluency [%] at the start of the assay. For the detection of mitochondrial ROS production, cells were incubated with 5 µM red MitoSOX™ mitochondrial superoxide indicator (Invitrogen, #M36008) in PBS with 2% FCS. ATP (2 mM) or antimycin A (AA, 10 µM) were added as indicated.

### 2.14 Agilent Seahorse XF Cell Mito Stress and Glycolysis Stress Test

50,000 BMMs (150 µl/well) were seeded in Seahorse 96-well cell culture plates (Agilent, #103793–100) the day before the assay. Cells were left untreated or treated for 4 h with LPS (100 ng/ml). The Mito Stress Test (Agilent, #103015–100) and Glycolysis Stress Test (Agilent, #103020–100) were performed according to the recommendations in the manufacturer’s protocol in a Seahorse XFe96 Analyzer (Agilent). OCR and ECAR measurements were normalized to cell count as determined in a Cytation 1 Cell Imaging Reader (BioTek) from brightfield images taken after the assay using the Gen5 software (Version 3.14) as described previously (47).

### 2.15 Quantification of mitochondrial DNA copy number

Mitochondrial DNA (mtDNA) copy number was determined following the protocol by Quiros et al. (48). Briefly, DNA was extracted from 250,000 BMMs cultured in 0.5 ml of BMM media per well (12-well plate) using the DNA Mini Prep Plus kit (ZymoResearch, #D4069) as per the manufacturer’s instructions. Subsequently, qRT-PCR was employed to measure the gene expressions of *mt16S*, *mtND1*, and *Hk2*, utilizing primer sequences adapted from the referenced publication. The mitochondrial DNA (mtDNA) copy number was calculated as the ratio between mtDNA content (mean expression of *mt16S* and *mtND1*) and nuclear DNA (nDNA) represented by *Hk2* expression.

### 2.16 Flow cytometry

10^6^ BMMs (2 ml/well) were seeded in 6-well plates. On the next day, cells were detached by gently scraping the wells with a cell scraper (TPP^®^), resuspended in 2% FCS-containing PBS, stained for 45 min at 37°C with 0.1 µM MitoTracker Green FM and Deep Red FM (Invitrogen, #M22426 and #M7514 respectively), and washed with 2% FCS-containing PBS. Flow cytometry measurements were performed using an LSRFortessa™ (BD Biosciences) operated by FACS Diva 8.0.1 (BD Biosciences). In each measurement, at least 50,000 events were recorded. MFI of unstained control cells was subtracted from corresponding stained cells, and background-subtracted MFI values for WT cells were set as 100%. The pseudocolor plots were generated utilizing FlowJo 10.10.0 software.

### 2.17 Cell migration and proliferation assays

Cell migration and proliferation capacity were measured using the Incucyte^®^ S3 Live-Cell Analysis System (Essen BioScience) as described previously (47, 49).

To assess migration capacity, L929 cells (ATCC: CCL-1™) were seeded (50,000 cells per well in 100 µl medium) in 96-well ImageLock™ plates (Essen BioScience, #BA-04856) and grown to full confluency overnight. Cells were cultured as described above. On the next day, cells were treated for 2 h with 5 µg/ml mitomycin C and washed with PBS before every well was scratched uniformly using the WoundMaker™ (Essen BioScience). Detached cells were washed away with PBS before BMM-conditioned media were added to the cells (100 µl/well). Plates were immediately placed into the Incucyte^®^ S3 Live-Cell Analysis System (Essen BioScience) and imaged for relative wound closure [%] at indicated time points.

To assess proliferation capacity, L929 cells were seeded (5,000 cells/well, 100 µl) and incubated overnight. On the next day, the medium was replaced by BMM-conditioned medium (100 µl/well). Cell confluency was measured over time in the Incucyte^®^ S3 Live-Cell Analysis System (Essen BioScience) and normalized to starting confluency in each well.

### 2.18 Gelatin zymography

MMP activity was measured as previously described (50). In brief, BMM-conditioned media were loaded onto a 10% SDS-acrylamide gel containing 1 mg/ml gelatin and electrophoresis was performed. Gels were washed in 2.5% Triton-X 100 to remove SDS and after a 72-hour incubation at 37°C in a buffer conducive to enzyme activity (50 mM Tris, 5 mM CaCl_2_ and 0.02% Brij-35), the gel was stained with Coomassie solution. After brief incubation in destaining solution, specific MMP activity, indicating gelatin degradation as clear bands on a dark background, was assessed based on their molecular weights. Conditioned media from each individual biological replicate was measured in technical duplicates. Gels were scanned with an Odyssey^®^ CLx Infrared Imaging System (LI-COR Biosciences) and inverted to enhance visibility, presenting bands in black against a white background. Signals were quantified using the Odyssey^®^ Image Studio software (Version 5.2).

### 2.19 Data representation and statistics

Visualization, advanced analysis, and all statistical tests were performed in Origin Lab 2018b. Data are presented as means ± standard error of the mean (bars). Each dot within the bar graphs represents one independent cell preparation. The mean of two groups was tested for statistical significance using the Mann-Whitney *U*-test. Statistical significance across all genotypes (mean of 3 groups) was assessed through one-way ANOVA (for single time point comparisons) and two-way ANOVA (for multiple time points) both followed by Bonferroni *post-hoc* tests.

## 3 Results

### 3.1 Differentially expressed genes in WT, GILZ KO, and GILZ-overexpressing macrophages

**Figure 1:**
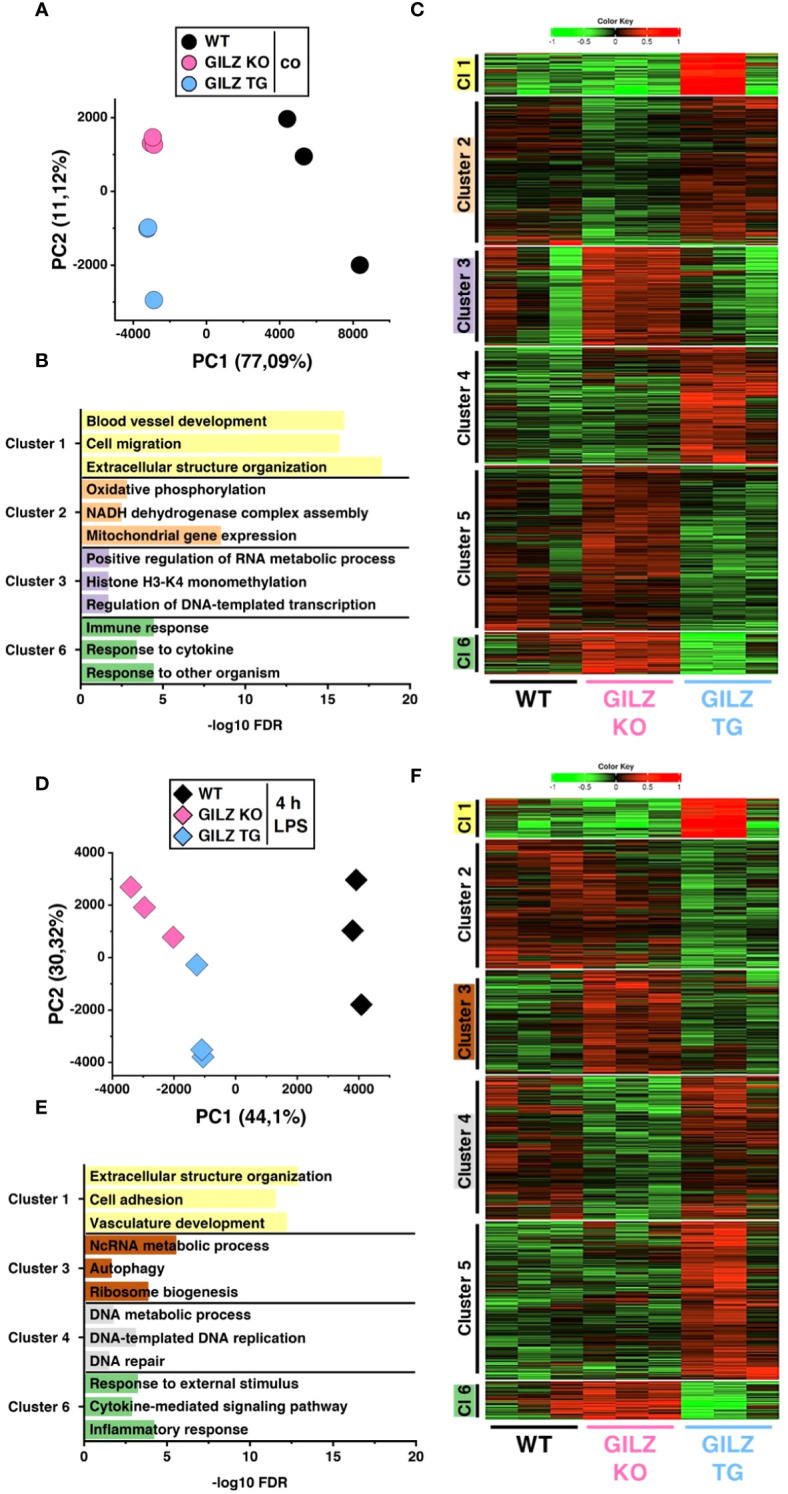
Differentially Expressed Genes in WT, GILZ KO, and GILZ-Overexpressing Macrophages. **(A, D)** Principal component (PC) analysis of the samples used in C and F, respectively (input data = *counts per million*). Each dot represents one independent biological preparation. k-Means unsupervised clustering of all differentially expressed genes from DeSeq2 analysis (input data = *transcripts per million*) using iDEP 1.12 in untreated **(C)** and LPS-treated **(F)** BMMs of n = 3 WT, GILZ KO, and GILZ TG mice per group. 1 (red) = maximum upregulation, -1 (green) = maximum downregulation. **(B, E)** Selected GO terms for biological processes of the clusters (Cl) shown in C and F, respectively. Not all clusters are characterized by distinct GO term profiles. FDR, false discovery rate.

We conducted RNA-Seq with wild-type (WT), GILZ knockout (GILZ KO), and GILZ-overexpressing (GILZ TG) bone marrow-derived macrophages (BMMs), both under untreated conditions and following a 4 h LPS treatment. The RNA-Seq data, analyzed using the DESeq2 approach for the WT vs. KO, WT vs. TG, and KO vs. TG comparisons, revealed 3,286 differentially expressed genes (DEGs, *p* < 0.05) in untreated cells. This number increased to 3,716 with LPS treatment, encompassing all three comparisons (**Supplementary File S1**). Principal component analysis (PCA) was employed to explore the variance in gene expression among samples. The obtained data revealed distinct grouping patterns based on the genotype, underscoring that observed gene expression variations are closely associated with differences in GILZ abundance (**Figures 1A, D**). Next, we employed iDEP, a tool designed for identifying patterns in gene expression across all samples. The tool utilized *transcripts per million* (TPM) data of the DEGs, independently for untreated and LPS-treated cells, to conduct k-Mean hierarchical clustering. In **Figures 1B, E**, representative Gene Ontology (GO) terms for biological processes from hierarchical clustering are linked to their corresponding false discovery rates (FDRs), providing insights into the functional categories influenced by GILZ expression (**Supplementary File S2**). Genes involved in the inflammatory response of macrophages (Cluster 6) showed differential expression under both untreated and LPS-treated conditions. Moreover, two out of three GILZ TG samples exhibited a strong induction of genes associated with vasculature development, cell mobility, and extracellular structure organization (Cluster 1).

### 3.2 Enhanced phagocytic and antibacterial activity in GILZ-overexpressing macrophages

**Figure 2:**
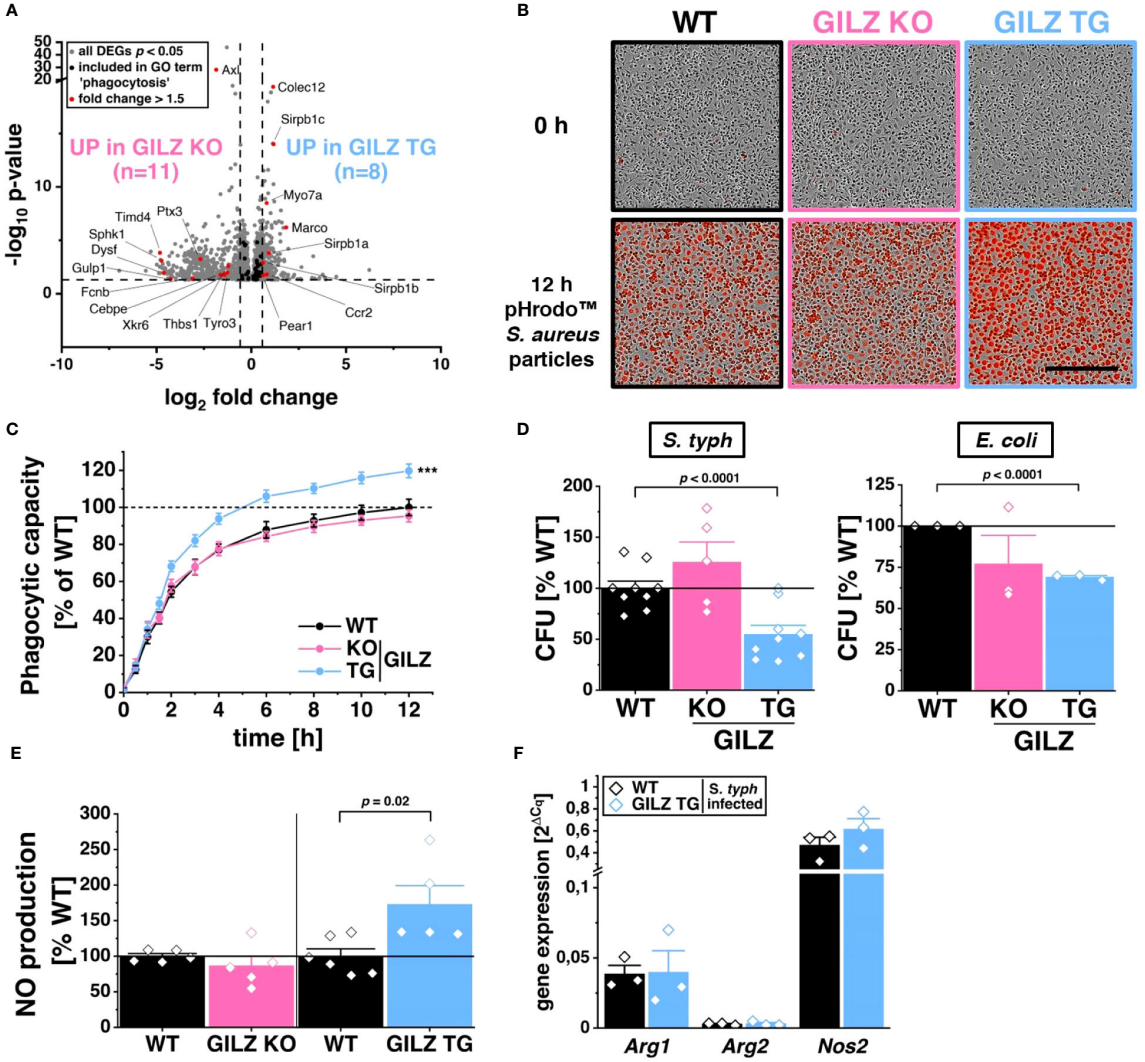
Enhanced Phagocytic and Antibacterial Activity in GILZ-Overexpressing Macrophages. **(A)** Volcano plot of all differentially expressed genes (DEGs, *p* < 0.05, shown in grey) from untreated BMMs comparing GILZ KO and GILZ TG. Shown in black are all genes annotated in the GO term ‘phagocytosis’ (GO:0006909), from which those with a fold change < 0.67 or > 1.5 are shown in red (n= number of red genes for GILZ GO and TG). **(B)** Representative brightfield and red fluorescence channel images and phagocytic capacity **(C)** from live cell imaging with the Incucyte^®^ S3 system after the addition of red pHrodo™ particles on untreated BMMs (n = 10 mice per group) relative to WT (set to 100%). Scale bar: 350 µm. **(D)** Colony-forming units (CFU) relative to WT (set to 100%) after *S. typhimurium* (n = 5–9 mice per group) and *E*. *coli* (n = 3 mice per group) infection assays. **(E)** Relative NO production from LPS/IFN-γ-treated BMMs (n = 5–6 mice per group) measured by Griess assay relative to WT (set to 100%). **(F)** Quantitative RT-PCR detection of *Arg1*, *Arg2*, and *Nos2* in BMMs infected with *S. typhimurium* for 6 h (n = 3 mice per group). *p* values were generated from one-way ANOVA comparison between groups for single time points and two-way ANOVA comparison for multiple time points both followed by Bonferroni *post-hoc* tests. ***p < 0.0001.

RNA-Seq analysis identified 55 differentially expressed genes associated with the GO term ‘phagocytosis’ (GO:0006909) in both GILZ KO and GILZ TG macrophages. Notably, these DEGs exhibited a balanced distribution of upregulation and downregulation in both cell types (**Figure 2A**). To elucidate the functional implications of these transcriptional changes, we conducted live cell imaging of macrophages after the addition of pHrodo™ particles, emitting red fluorescent light upon engulfment (**Figure 2B**). Quantitative analysis revealed that GILZ-overexpressing macrophages exhibited a higher phagocytic capacity over 12 hours compared to WT macrophages, with a noticeable increase beginning at the 2-hour time point (**Figure 2C**). Given the observed alterations in phagocytosis, we investigated antibacterial activity in BMMs against *S. typhimurium* and *E. coli*. Notably, GILZ-overexpressing macrophages exhibited not only increased phagocytosis but also enhanced bacterial clearance against both tested pathogens (**Figure 2D**). In this context, GILZ KO cells exhibited a tendency of attenuated phagocytic capacity, albeit not reaching statistical significance, and results regarding bacterial clearance were inconclusive. Consequently, our focus shifted to GILZ-overexpressing macrophages, aiming to investigate whether the observed effects stem from increased NO production, which is one of the main antibacterial factors in murine macrophages (51). Indeed, GILZ TG macrophages demonstrated elevated NO production when polarized to M1 (**Figure 2E**). In M1 macrophages, inducible nitric oxide synthase (NOS2) is the main producer of NO, while arginases catalyze arginine hydrolysis to ornithine and urea, competing with NO synthases for arginine (52). Arginase 1 (ARG1) inhibits NO production and acts as an M2 marker, whereas Arginase 2 (ARG2) is involved in reducing M1 activation through metabolic reprogramming (53, 54). Upon infection, there was a tendency for increased expression of *Nos2* but neither altered expression of *Arg1* nor *Arg2* (**Figure 2F**).

### 3.3 Protection from pyroptosis in GILZ-overexpressing macrophages

**Figure 3:**
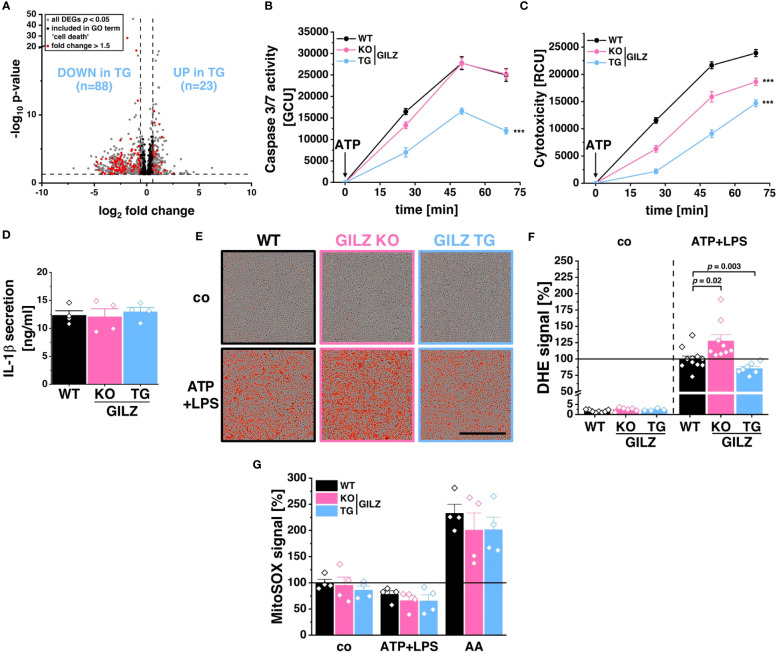
Protection from Pyroptosis in GILZ-Overexpressing Macrophages. **(A)** Volcano plot of all differentially expressed genes (DEGs, *p* < 0.05, shown in grey) from untreated BMMs comparing GILZ KO and GILZ TG. Shown in black are all genes annotated in the GO term ‘cell death’ (GO:0008219), from which those with a fold change < 0.67 or > 1.5 are shown in red and counted, corresponding to the indicated n number. Normalized green (Caspase-3/7 activity) **(B)** and red (Cytotoxicity) **(C)** signal of BMMs upon ATP treatment measured by live cell imaging with the Incucyte^®^ S3 system of BMMs pre-treated with LPS (n = 6 mice per group). **(D)** IL-1β secretion measured by ELISA of ATP/LPS-treated BMMs (n = 4 mice per group). **(E)** Representative brightfield and red fluorescence channel images and **(F)** DHE signal from live cell imaging with the Incucyte^®^ S3 system in untreated BMMs (co) and 30 min after the addition of ATP on LPS-treated BMMs with cell culture media containing DHE relative to stimulated WT (set to 100%, n = 4–9 mice per group). Scale bar: 700 µm. **(G)** MitoSOX™ signal from live cell imaging with the Incucyte^®^ S3 system in untreated BMMs (co), 30 min after the addition of either ATP on LPS-treated BMMs (ATP+LPS) or Antimycin A (AA) relative to WT (set to 100%). *p* values were generated from one-way ANOVA comparison between groups for single time points and two-way ANOVA comparison for multiple time points both followed by Bonferroni *post-hoc* tests. ***p < 0.0001.

RNA-Seq analysis revealed 111 differentially expressed genes included in the ‘cell death’ GO term (GO:0008219), with 88 downregulated in GILZ TG macrophages (**Figure 3A**). This downregulation pattern prompted the hypothesis of a protective mechanism against specific forms of cell death. Upon inflammasome activation by ATP+LPS treatment, GILZ TG macrophages exhibited a significant reduction in caspase-3/7 activation compared to WT (**Figure 3B**). Simultaneously, our evaluation of cell viability revealed enhanced cell survival in GILZ-overexpressing macrophages, as indicated by a substantial decrease in the cytotoxicity signal, originating from compromised plasma membrane integrity and a subsequent increase in fluorescence upon binding to DNA (**Figure 3C**). Given that ATP+LPS treatment induces pyroptotic cell death through caspase-1 activation, responsible for processing IL-1β, we explored IL-1β secretion under these conditions and found no significant differences between genotypes (**Figure 3D**). This led us to conclude that GILZ overexpression selectively influences specific facets of the signaling cascade following ATP+LPS treatment in macrophages. Given the pivotal role of reactive oxygen species (ROS) in promoting NLRP3 inflammasome activation and subsequent cell death, we investigated ROS production using DHE dye with live cell microscopy (**Figure 3E**). Notably, GILZ TG macrophages exhibited a significant reduction to 80% in ROS production, while GILZ KO macrophages displayed an increased production to 130% compared to WT macrophages (**Figure 3F**). Upon inflammasome activation, mitochondria are known to be both sources and targets of reactive oxygen species (55). We tracked mitochondrial ROS (mitoROS) production under ATP+LPS treatment using mitoSOX fluorescence, a ROS dye specific for mitochondria. Intriguingly, across all genotypes, we observed a reduction of mitoROS production to 60% upon ATP+LPS treatment (**Figure 3G**), suggesting that mitoROS production may not be directly linked to cell viability. Additionally, we induced mitoROS production using Antimycin A (AA) as a positive control, and as expected, it elicited a substantial increase (*p <* 0.0001) in mitoROS across all genotypes. Interestingly, we observed a trend of diminished mitoROS levels in GILZ TG macrophages under untreated conditions. This observation piqued our interest and warranted a characterization of mitochondria in these macrophages.

### 3.4 Lower respiration rate in GILZ-overexpressing macrophages

**Figure 4:**
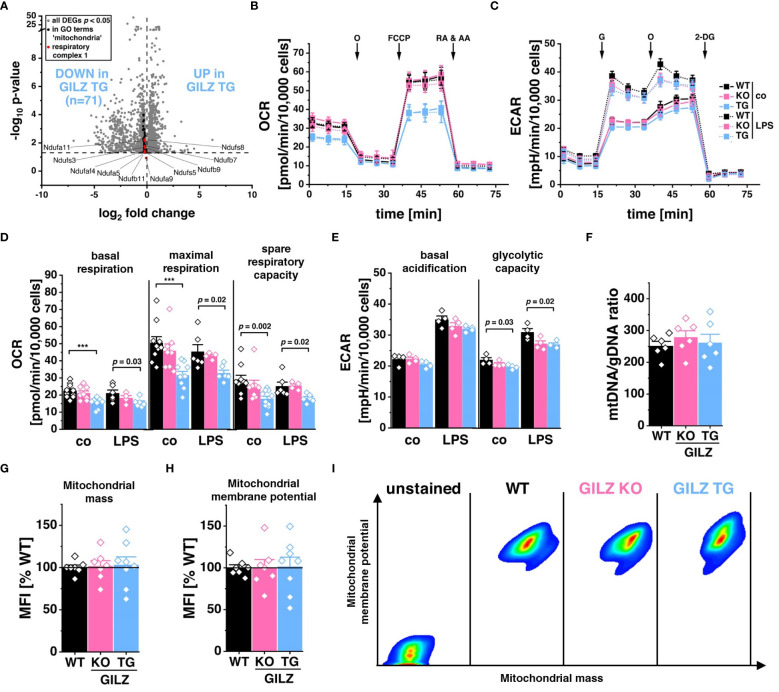
Lower Respiration Rate in GILZ-Overexpressing Macrophages. **(A)** Volcano plot of all differentially expressed genes (DEGs, *p* < 0.05, shown in grey) from untreated BMMs comparing GILZ KO and GILZ TG. Shown in black are all genes annotated to GO terms related to mitochondria (GO:0140053, GO:0006119, GO:0010257), from which those encoding for respiratory complex I are shown in red and counted, corresponding to the indicated n number. **(B, D)** Oxygen consumption rate (OCR) during the mitochondrial stress test and **(C, E)** extracellular acidification rate (ECAR) during the glycolysis stress test in untreated (co) and LPS-treated BMMs measured with the Seahorse XFe96 Analyzer (n = 4–6 mice per group). O, oligomycin; RA & AA, rotenone & antimycin A; G, glucose; 2-DG, 2-deoxy-d-glucose **(F)** Quantitative RT-PCR detection of mtDNA copy number in BMMs (n = 6 mice per group). Flow cytometric measurement of **(G)** mitochondrial mass, and **(H)** mitochondrial membrane potential in BMMs relative to WT (set to 100%, n = 7–8 mice per group), and **(I)** pseudocolor plots of representative samples. *p* values were generated from one-way ANOVA comparison between groups followed by Bonferroni *post-hoc* test. ***p < 0.0001.

RNA-Seq analysis unveiled the significant downregulation of 71 genes associated with Gene Ontology terms related to ‘mitochondria’ (GO:0140053, GO:0006119, GO:0010257), corresponding to those depicted in **Figure 1C** as Cluster 2 and all specific to GILZ-overexpressing macrophages (**Figure 4A**). Particularly, among these genes, 15 of the 59 encode for subunits of the NADH:ubiquinone oxidoreductase, the key component of Complex I in the electron transport chain (GO:0010257, FDR = 3.4 x 10–^3^). Motivated by these transcriptomic findings, we subsequently examined the functional implications by assessing the oxygen consumption rate (OCR) in these cells (**Figure 4B**). Interestingly, our analysis unveiled a significant reduction not only in basal respiration but also in maximal respiration and spare respiratory capacity in GILZ-overexpressing macrophages, both under untreated conditions and following LPS treatment (**Figure 4D**). When mitochondrial respiration is compromised, it often leads to compensatory adjustments in glycolytic activity (47, 56, 57). Therefore, to elucidate the glycolytic response, we performed a glycolysis stress test focusing on extracellular acidification rate (ECAR) (**Figure 4C**). Our results demonstrated a slight reduction of glycolytic capacity in GILZ TG macrophages (**Figure 4E**). Notably, these metabolic alterations are not merely a consequence of a decrease in mitochondrial content, as indicated by equivalent mitochondrial DNA (mtDNA) content (**Figure 4F**), comparable mitochondrial mass (**Figure 4G**), and unchanged mitochondrial membrane potential (**Figures 4H, I**) in GILZ TG macrophages.

### 3.5 Elevated MMP activity in GILZ-overexpressing macrophages

**Figure 5:**
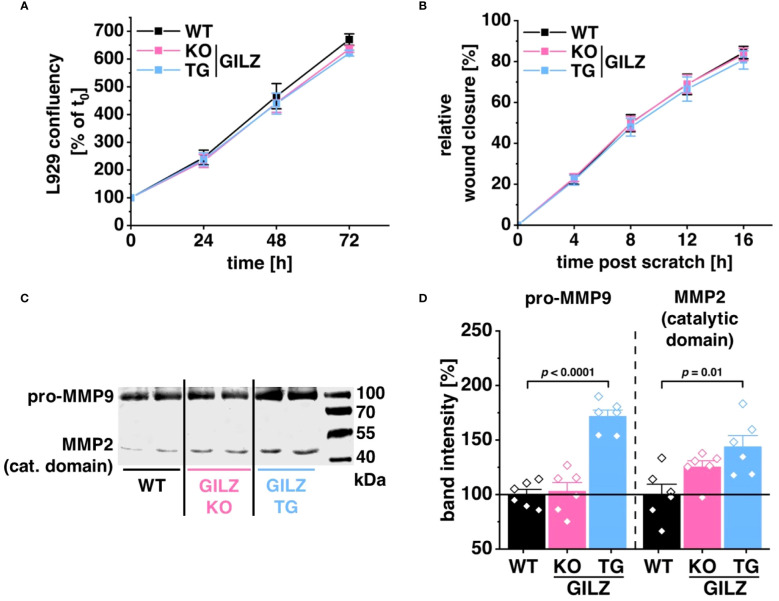
Elevated MMP Activity in GILZ-Overexpressing Macrophages. **(A)** Proliferation of L929 cells in conditioned media from untreated BMMs, measured as a relative increase in cell confluency over time using live cell imaging with the Incucyte^®^ S3 system (n = 8 mice per group). Data are normalized to the starting confluency of each well (set to 100%). **(B)** Migration of mitomycin C-treated L929 cells in response to wounding, calculated as an increase in cell confluency in the wounded area over time using live cell imaging with the Incucyte^®^ S3 system (n = 6 mice per group). **(C)** Representative gel scan and **(D)** relative quantification of MMP2 and pro-MMP9 activity relative to WT (set to 100%) measured by gelatin zymography (n = 6 mice per group). *p* values were obtained from one-way ANOVA comparisons between groups followed by Bonferroni *post-hoc* test.

RNA-Seq analysis disclosed 224 DEGs in untreated GILZ-overexpressing macrophages and 171 DEGs in LPS-treated cells associated with cell movement, wound healing, and extracellular structure organization forming Cluster 1 upon k-mean unsupervised clustering of TPM values of DEGs (p < 0.05) from DESeq2 analysis in all three comparisons (**Figures 1C, F**). Notably, among these DEGs forming Cluster 1, 80 genes were shared between untreated and LPS-treated BMMs. To discern the functional implications of these transcriptomic findings, we investigated their impact employing the murine fibroblast cell line L929. Surprisingly, cell proliferation (**Figure 5A**) or migration in response to wounding (**Figure 5B**) was unaffected by the macrophage genotype when L929 cells were exposed to media conditioned by WT, GILZ KO, or GILZ TG macrophages. Despite these outcomes, the RNA-Seq data, highlighting genes related to the extracellular matrix, prompted an investigation of MMP activity in the conditioned media using gelatin zymography (**Figures 5C, D**). The results unveiled heightened pro-MMP9 and MMP2 activity in media conditioned by GILZ TG macrophages. These findings correlate with the increased gene expression observed in the RNA-Seq data for these enzymes.

## 4 Discussion

In recent years, the exploration of therapeutic GILZ use has extended beyond its well-known anti-inflammatory effects, gaining interest for its potential pro-resolving actions. Traditionally, in macrophages, GILZ is postulated to confer an anti-inflammatory phenotype to monocytes and macrophages (58). It is recognized for inhibiting the expression of proinflammatory molecules, preventing toll-like receptor 2 production, and impeding NF-kB function (59). Moreover, GILZ can be induced by the natural product curcumin, contributing to its anti-inflammatory effects in macrophages (38). Additionally, GILZ plays a crucial role in modulating macrophage responses to LPS exposure; downregulation of GILZ is linked to heightened NF-κB and AP-1 activity, accompanied by increased pro-inflammatory cytokine production (36). Notably, M1-like macrophages from GILZ-deficient mice exhibit elevated expression of M1 markers and reduced expression of IL-10 compared to M1-like macrophages from wild-type mice (60). Consistent with these findings, our RNA-Seq data revealed that inflammatory pathways are upregulated in GILZ KO macrophages compared to GILZ-overexpressing macrophages both under control and inflammatory conditions.

Examining the resolution of inflammation, GILZ emerges as a pivotal mediator of the anti-inflammatory and immunomodulatory effects of GCs (15, 16). Moreover, GILZ demonstrates its potential in effectively resolving detrimental inflammation by regulating leptin production in osteoarthritis synovial fibroblasts (61), safeguarding myocardial cells (29), reducing spinal cord tissue damage and leukocyte infiltration (31), and inhibiting leukocyte recruitment in liver fibrosis (62). Notably, during the resolution phase of LPS-induced pleurisy, GILZ expression exhibits an increase, particularly in macrophages displaying resolving phenotypes, reinforcing its remarkable resolving actions (63). Nevertheless, the regulation of GILZ in macrophages appears to be intricate and contingent on the context. Various factors, including hypoxia (21), IL-10 (59), and proinflammatory cytokines (23), can influence the expression of GILZ in macrophages. Interestingly, GILZ levels are stabilized in macrophages characterized as LPS tolerant (36). Furthermore, the absence of GILZ has been linked to macrophage aging, which is linked to a low-grade inflammatory condition (46).

### 4.1 Antibacterial activity

In addition to its recognized anti-inflammatory effects, GILZ has demonstrated accompanying benefits in the context of bacterial infections, broadening its potential therapeutic applications. To characterize these additional effects, we conducted investigations involving GILZ KO and GILZ TG macrophages, aiming to uncover mechanistic insights through transcriptomic data analysis. Our study demonstrates enhanced phagocytic activity and increased killing efficacy of *S. typhimurium* [an intracellular pathogen (64)] and *E. coli* [using a non-invasive strain (65)] by GILZ-overexpressing macrophages, potentially mediated by an increased capacity to produce nitric oxide (NO). In this context, it is noteworthy to examine analogous observations in existing literature. Notably, transgenic mice with heightened GILZ expression specifically in monocytes and macrophages exhibited lower frequencies of inflammatory monocytes, reduced plasma levels of inflammatory cytokines, and diminished blood bacterial counts when experiencing sepsis (32). This upregulation of GILZ in monocytes and macrophages improved their phagocytic capacity in *in vivo* assays and heightened the survival rates of septic mice. Additionally, TAT-GILZ, a fusion protein combining the cell-penetrating peptide TAT with GILZ, was found to induce the release of CCL2, facilitating monocyte/macrophage recruitment through the CCR2 receptor and resulting in accelerated resolution of *E. coli-*induced neutrophilic inflammation, increased peritoneal numbers of monocytes/macrophages, enhanced apoptosis/efferocytosis counts, and improved bacterial clearance through phagocytosis (35). In the context of pneumococcal pneumonia, TAT-GILZ treatment reduced neutrophilic inflammation, enhanced macrophage efferocytosis, and improved bacterial clearance (34). Our findings in this study differ from our previous findings, which indicated enhanced phagocytosis and killing efficiency in GILZ KO macrophages (39). However, it is essential to note that different models were employed in the two studies. Specifically, phagocytic capacity was assessed using latex beads, emphasizing the material’s impact on phagocytic activity, as highlighted by (66). Moreover, the measurements of NO production were conducted using a different cell type, and the infection conditions differed from those in our prior study. However, the precise molecular mechanisms through which GILZ exerts dual effects, enhancing antibacterial clearance and resolving inflammation effects, require further elucidation.

Contrary to GILZ, GCs have a negative impact on the antibacterial function of macrophages. They inhibit the phagocytic activity of macrophages, leading to an increase in the severity of bacterial infections such as tuberculosis (67). Furthermore, GCs have been found to directly upregulate the expression of DPP4, leading to the mobilization and enhanced activity of macrophages, which may exacerbate rather than mitigate macrophage-dominated inflammatory disorders in the context of GC therapy (68). GCs impair phagocytosis against Crohn’s disease-associated adherent-invasive *E. coli* (AIEC) (69). Moreover, GCs have been demonstrated to suppress antimicrobial autophagy and nitric oxide production in macrophages, thereby facilitating mycobacterial survival (70).

### 4.2 Pyroptosis

Considering our discovery that GILZ overexpression protects macrophages from pyroptosis, we propose a potential regulatory role for GILZ in the pyroptotic cell death pathway. Pyroptosis is a lytic form of programmed cell death triggered by the detection of pathogens or host-derived danger signals. Gasdermin-D (GSDMD) functions as the central pyroptotic substrate for inflammatory caspases. This process results in the formation of pores, causing cell swelling until membrane rupture occurs. Consequently, this rupture leads to the release of inflammatory cytokines such as IL-1β, IL-6, and IL-18 into the extracellular space (71). In our study, we observed that GILZ expression did not impact IL-1β secretion upon inflammasome activation, suggesting that GILZ may not influence cytokine processing by caspases within the pyroptosis cascade. Consequently, we redirected our focus to another well-established host-derived danger signal in this context. This signal centers on the detection of reactive oxygen species (ROS), known to induce the assembly of either noncanonical or canonical inflammasomes (72). Increasing levels of ROS by an upregulated NADPH oxidase have been shown to induce pyroptotic cell death (73). Recently, Devant and colleagues showed in macrophages that the terminal step in the pyroptosis pathway, namely pore formation, relies on ROS to oxidize the caspase cleaved GSDMD (74). Given the observed reduction in ROS levels in GILZ-overexpressing macrophages, our study suggests a potential interference by GILZ with ROS production, specifically after inflammasome assembly at a later stage in the pyroptotic cascade. As the exact origins of ROS production during pyroptosis and potential interaction partners for GILZ remain unclear, further investigations are needed. Notably, supporting our findings, previous research has established that decreased ROS levels confer protection against ATP-LPS-induced cell death in BMMs (75). Contrary to the common association of ROS generation with mitochondrial dysfunction, our study revealed no increase in mitochondrial ROS. Nonetheless, it is noteworthy that mitochondrial ROS have been identified as crucial contributors to pyroptosis via GSDMD oxidation (76). Additionally, TRAF3 was shown to amplify mitochondrial ROS production and pyroptosis through ULK1 ubiquitination (77). Ceramide-mediated regulation of mitochondrial ROS was observed to connect ROS, NLRP3–caspase-1 inflammasome activation, and pyroptosis in cancer cells (78). Furthermore, scavenging mitochondrial ROS effectively mitigated pyroptosis and IL-1β secretion in the context of hyperglycemia- and periodontitis-related pyroptosis (79).

With a focus on their potential to activate the NLRP3 inflammasome and induce pyroptosis, the detrimental effects of GCs have been extensively studied in various cellular and disease models. Studies on muscle cells suggest that GCs may induce pyroptosis through the NLRP3/GSDMD pathway, implying a role in muscle atrophy (80–82). Chronic GC exposure primes microglia to pro-inflammatory stimuli, inducing *Nlrp3* mRNA in the hippocampus, suggesting a link between GCs and neuroinflammation (83–85). In macrophages, sensitivity to GCs concerning the NLRP3 inflammasome appears model dependent. GCs enhance ATP-dependent NLRP3 expression in cultured human monocytes/macrophages, promoting pro-inflammation (86, 87). Conversely, under LPS stimulation, an inhibitory role is suggested in cultured murine macrophages (88). This once again emphasizes the need to explore alternative therapeutic options such as GILZ.

### 4.3 Respiration and mitochondria

Mitochondria are known to act as a signaling platform in macrophages, linking energy metabolism and macrophage polarization upon activation (89). In our study, we found that macrophages overexpressing GILZ have significantly less mitochondrial oxidative phosphorylation capacity, which is linked to a downregulation of numerous genes encoding for complex I of the electron transport system. This contrasts with findings in cancer cells where GILZ has been implicated in enhancing mitochondrial oxidative phosphorylation, leading to increased susceptibility to death induced by mitochondrial pro-oxidants (90, 91). These contrasting effects underscore the context-dependent role of GILZ in mitochondrial function and warrant further investigation into its diverse mechanisms of action in different cellular contexts.

GCs have been shown to have a significant impact on mitochondrial function in macrophages. Mitochondrial GRs alter the expression of genes involved in oxidative phosphorylation, which can affect the activity and abundance of key enzymes in the electron transport chain, leading to changes in ATP production (92). Mitochondrial function is indispensable for GCs to exert their effects, as evidenced by studies demonstrating that the inhibition of complex V abolishes dexamethasone-mediated upregulation of *Gilz* (93). Chronic GC treatment of cortical neurons has been shown to reduce mitochondrial oxidation (94). Altogether, this leads us to speculate that GILZ may exert effects on macrophages similar to its primary inducers, GCs, suggesting potential molecular interactions that require elucidation in future studies.

### 4.4 Tissue remodeling

The versatility of macrophages allows them to participate across all phases of inflammation, from instigating the pro-inflammatory response to facilitating tissue repair post-injury. Considering this, we could identify a new aspect of GILZ’s role in inflammatory conditions, particularly in its promotion of pro-resolving actions, such as the activation of matrix metalloproteinases (MMP) when GILZ is overexpressed in macrophages. The secretion of MMP9 and MMP2 by macrophages has been widely recognized as playing a crucial role in wound healing. These metalloproteinases are involved in the degradation and remodeling of the extracellular matrix (ECM), which is essential for various stages of the wound healing process (95–97). We detected pro-MMP9 via gelatin zymography. *In vivo*, macrophage-derived pro-MMP9 can be activated by MMP-3 or other MMPs (98). Although these gelatinase pro-forms are typically latent and catalytically inactive in the absence of their activating enzymes (99), they become activated during zymography due to the presence of the denaturing agent SDS (100). Overexpression of MMP9 in macrophages improves diastolic physiology and cardiac wound healing after myocardial infarction (101).

The effects of GCs on tissue repair extend beyond the activation of GRs but remain poorly understood (102). Findings in peripheral blood mononuclear cells suggest that GC-mediated monocyte/macrophage-specific induction of ADAMTS2, a secreted metalloproteinase involved in the processing of procollagen to collagen, may play a crucial role in the resolution of inflammation and wound repair (103). The precise mechanism by which GILZ might regulate MMP secretion in macrophages remains unclear and represents a novel area of investigation in the field.

## Data availability statement

Raw and processed data from the RNA-sequencing generated in the study were deposited in the Gene Expression Omnibus (GEO) database under the accession code GSE254137. Other original data in the present study are available from the corresponding author upon request.

## Ethics statement

The animal study was approved by the Landesamt für Verbraucherschutz, Saarbrücken, Germany. The study was conducted in accordance with the local legislation and institutional requirements.

## Author contributions

TL: Conceptualization, Data curation, Formal analysis, Funding acquisition, Investigation, Methodology, Validation, Visualization, Writing – original draft, Writing – review & editing. HS: Formal analysis, Investigation, Writing – review & editing. GG: Formal analysis, Software, Writing – review & editing. SM: Investigation, Writing – review & editing. JW: Funding acquisition, Project administration, Resources, Writing – review & editing. CL: Resources, Writing – review & editing. BD: Conceptualization, Funding acquisition, Methodology, Supervision, Writing – review & editing. JH: Conceptualization, Data curation, Funding acquisition, Methodology, Supervision, Validation, Writing – review & editing. AK: Conceptualization, Funding acquisition, Methodology, Project administration, Resources, Supervision, Writing – review & editing.
